# Duckweeds: from fundamental biology to a sustainable plant chassis for biotechnology

**DOI:** 10.1007/s44307-026-00110-1

**Published:** 2026-04-16

**Authors:** Gui-Min Yin, Lin Yang, Sha Li, Yan Zhang

**Affiliations:** 1 College of Life Sciences, Nankai University, Tian’jin, China; 2 College of Life Sciences, Tianjin Normal University, Tian’jin, China; 3 College of Life Sciences, Shandong Agricultural University, Tai’an, China

**Keywords:** Duckweed, Plant chassis, Molecular farming, Sustainable biotechnology, Omics

## Abstract

Duckweeds (*Lemnaceae*), the smallest and fastest-growing flowering plants, have emerged as a transformative platform for sustainable biotechnology. This review synthesizes recent advances that underpin their potential as a next-generation plant chassis. We discuss duckweed's unique biology, characterized by reductive evolution, extreme phenotypic plasticity, and a simplified epigenome that favors transgene expression. The decoding of its minimalist genome, along with the establishment of efficient genetic tools including optimized transformation and CRISPR-Cas9 editing, enables precise genetic and metabolic engineering. While traditional uses in phytoremediation and animal feed validate its utility, duckweed's rapid growth in contained, soil-free culture and its edibility offer distinct advantages for molecular farming over established systems like tobacco. We highlight progress in engineering duckweeds to produce vaccines, therapeutic proteins, and high-value metabolites. To transition from proof-of-concept to an industrial workhorse, future efforts must focus on integrated omics databases, universal genetic toolkits, and scalable cultivation. Converging fundamental insights with synthetic biology principles positions duckweed as a versatile and powerful chassis for the bioeconomy.

## Introduction

Duckweeds (*Lemnaceae*), the smallest and fastest-growing flowering plants on Earth, have long been recognized for their roles in traditional agriculture and aquatic ecosystems. However, recent advances across multiple biological disciplines have catapulted these humble aquatic plants to the forefront of sustainable biotechnology. This review synthesizes the transformative progress positioning duckweed not merely as a biological curiosity, but as a powerful, next-generation plant chassis for the bioeconomy.

Building upon this foundation, the present review focuses specifically on the translation of duckweed from a model system to an engineered plant chassis for industrial biotechnology. While Acosta et al. (2021) provided an essential overview of duckweed biology and its historical context, our work is distinguished by its forward-looking, technology-centric perspective. We synthesize recent advances across three interconnected domains that collectively position duckweed as a next-generation production platform: (i) the explosion of high-quality omics resources (genomes, epigenomes, single-cell atlases) that have decoded the genetic basis of duckweed's minimalist biology; (ii) the rapid evolution of genetic tools, from foundational transformation protocols to CRISPR-Cas9 editing and high-throughput delivery methods; and (iii) the integration of these capabilities with practical applications, including molecular farming, metabolic engineering, and wastewater remediation. Critically, we provide a comparative analysis of duckweed against established plant chassis such as tobacco and microalgae, articulating its distinct advantages, namely contained aquatic cultivation, edibility, reduced lignin content, and simplified epigenome, which make it uniquely suited for scalable, contained biomanufacturing.

Furthermore, this review identifies and addresses key translational challenges that have not been systematically discussed elsewhere, including industrial-scale cultivation hurdles (oxygen dynamics, harvesting technologies), regulatory considerations for contained systems, and the need for integrated omics databases to enable predictive engineering. We conclude with a research roadmap that prioritizes the development of universal genetic toolkits, scalable bioreactor designs, and systems-level platforms to accelerate duckweed from proof-of-concept to industrial workhorse. In doing so, we aim to provide a comprehensive resource for researchers, bioengineers, and industry stakeholders seeking to harness duckweed's full biotechnological potential.

The journey begins with an exploration of duckweed's unique developmental biology and environmental plasticity, revealing a body plan shaped by reductive evolution for rapid, clonal propagation in aquatic environments and sophisticated adaptations like turion formation for stress resilience. Underpinning this phenotype is a streamlined genomic and epigenomic architecture (An et al. 2019; Dombey et al. 2025; Harkess et al. 2024), recently decoded by high-throughput omics, which reveals the genetic basis of its minimalism and unique features like reduced gene silencing pathways (Dombey et al. 2025; Harkess et al. 2024). Critically, the once-formidable barrier to genetic manipulation has been dismantled by the development of high-efficiency transformation and genome-editing tools, enabling precise engineering. While duckweed's traditional applications in phytoremediation, animal feed, and starch production validate its utility and safety, it is its convergence of rapid growth in contained culture, nutritional value, and genetic tractability that makes it an exceptional candidate for molecular farming (Thingujam et al. 2024). This review details how duckweed is being engineered to produce high-value biopharmaceuticals and metabolites, arguing that by integrating fundamental biological insights with synthetic biology principles, duckweed is poised to transition from a model organism to an industrialized, sustainable biofactory for diverse global challenges.

## The duckweed phenotype: developmental mastery and environmental plasticity

**Fig. 1:**
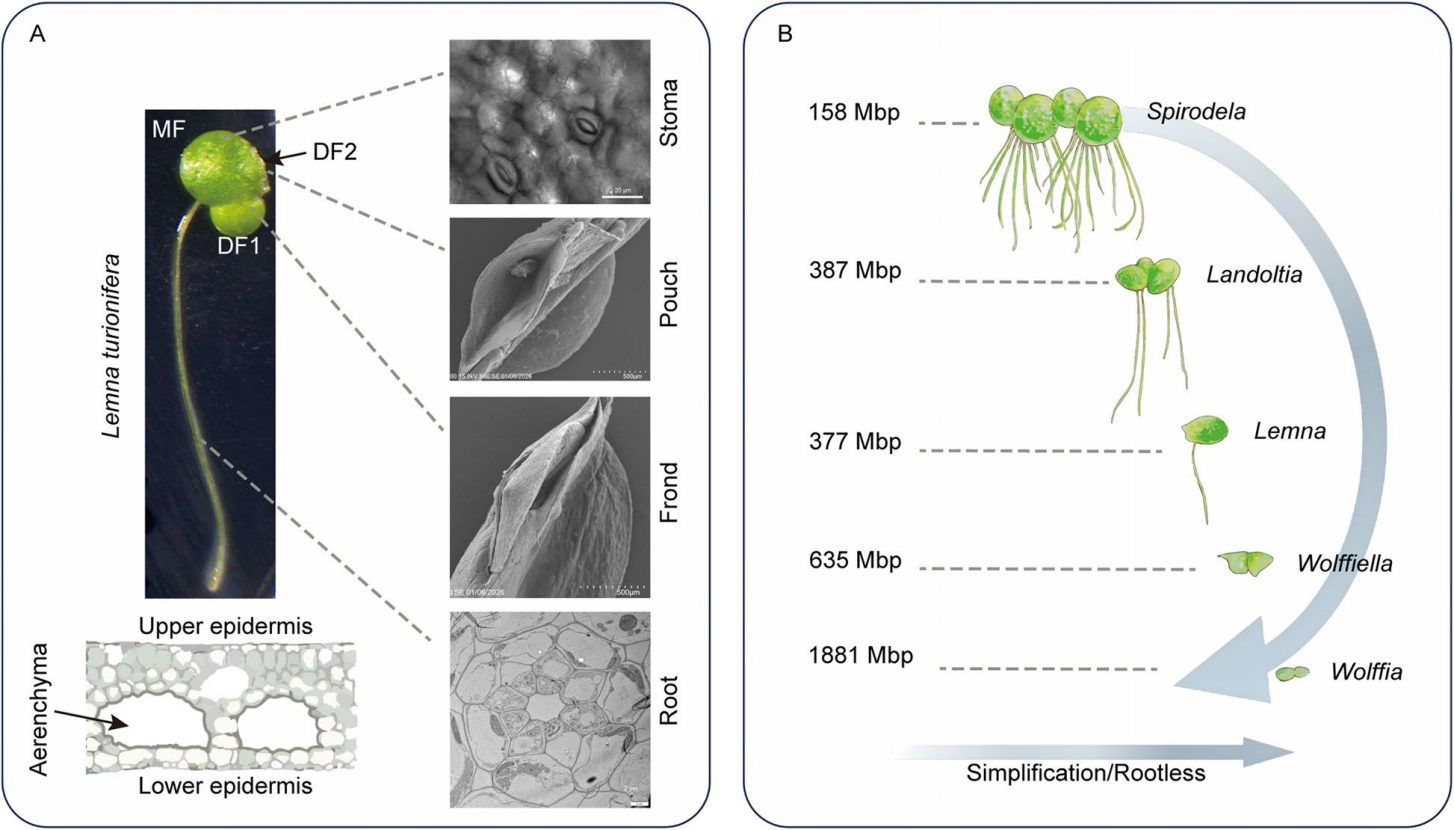
Body plan of duckweeds and their evolutionary reduction. **A** representative image of duckweed (e.g., *Lemna turionifera*). The overall morphology (left) is based on the observation under a stereomicroscope. The anatomical illustrations of key structures are on the right or bottom: stomata on the upper surface (by confocal microscopy), the meristematic pouch and frond (by scanning electron microscopy), root cross-section (by transmission electron microscopy), and the aerenchyma (in cartoon). **B** Schematic representation of the evolutionary reduction events across the *Lemnaceae* family, with corresponding genome sizes. The gradient proceeds from the structurally complex *Spirodela* (with multiple roots and larger fronds) to the highly reduced, rootless *Wolffia* (the smallest flowering plant). The labels on the left indicate the genome size of the representative strain sequenced: *Spirodela polyrhiza* 7498 (158 Mbp), *Landoltia punctata* 7260 (387 Mbp), *Lemna minor* 8623 (377 Mbp), *Wolffiella lingulata* 7655 (635 Mbp), and *Wolffia arrhiza* 8872 (1,881 Mbp). Note that there are intraspecific genome size variations in *Wolffia*. MF, mother frond; DF1, the first daughter frond; DF2, the second daughter frond; Mbp, million base pairs

Duckweeds (family *Lemnaceae*) represent a fascinating exception within the angiosperms (Fig. 1A-B). As the smallest and fastest-growing flowering plants, they exhibit a simplified morphology centered on a leaf-like structure called a frond, which can reproduce clonally every 1–3 days (Stomp 2005). This simplified architecture is the product of reductive evolution, a process by which ancestral land plants adapted to an aquatic lifestyle by progressively losing complex structures like extensive roots and vascular tissues (Acosta et al. 2021; Denyer et al. 2024). The family comprises five genera (*Spirodela*, *Landoltia*, *Lemna*, *Wolffiella*, *Wolffia*), forming an evolutionary gradient from the relatively complex, rooted *Spirodela* to the rootless, pinhead-sized *Wolffia* (Acosta et al. 2021). This neotenic lifestyle, in which the plant remains in a juvenile, vegetative state, is key to its rapid asexual proliferation and forms the biological foundation for its utility in biotechnology.

### Developmental processes in duckweed

#### Vegetative development and frond formation

Duckweed growth is driven by meristematic activity within one or two budding pouches located at the base of the frond. Detailed observation in *Lemna aequinoctialis* has shown that active cell proliferation in these pouches leads to the formation of daughter fronds (Yoshida et al. 2021). Despite this fundamental description, the precise cellular and molecular mechanisms enabling the extraordinarily rapid cycles of cell division, expansion, and abscission remain a major knowledge gap. Vesicular trafficking regulators, such as Rab GTPases, control the delivery of cell wall materials and membrane components to the sites of active growth (Nielsen 2020); their efficiency may be a key determinant of the rapid cell expansion and division rates observed in duckweed fronds (Yoshida et al. 2021) Similarly, growth-regulating transcription factors (GRFs) identified in duckweed genomes could provide crucial insights into this hyper-accelerated growth.

#### Flowering and sexual reproduction

Flowering is rare in duckweeds, but its induction is critical for breeding. Molecular components of the florigen pathway, including *FLOWERING LOCUS T* (*FT*) and *FLOWERING LOCUS D* (*FD*) genes, are conserved and functional in *Lemna* (Yoshida et al. 2021). Laboratory protocols to maximize flowering using specific day lengths, media, and supplements like salicylic acid have been optimized for several genera (Fourounjian et al. 2021). Recent discoveries of natural interspecific hybrids in the genus *Lemna* have revealed complex reproductive dynamics; while intraspecific hybrids are often fertile, many interspecific hybrids are male-sterile, and only established allotetraploid hybrids represent a true evolutionary breakthrough for sexual recombination (Lee et al. 2025).

#### Tissue and organ specialization

Recent studies have begun to decode specialized tissue development. A landmark study on the shoot aerenchyma (airspace) in *Spirodela polyrhiza* revealed that its spatial framework is established early through coordinated cell division and expansion. The connection of this network to the stomata involves programmed cell death (PCD). This entire process is governed by a precise spatiotemporal interplay of phytohormone signaling, involving ethylene, abscisic acid (ABA), and salicylic acid (Kim et al. 2024).

### Environmental responses and developmental plasticity

Duckweeds exhibit remarkable phenotypic plasticity in response to environmental cues, which directly impacts their development and biomass composition.

#### Nutrient responses

Nutrient availability is a primary growth regulator. While nutrient-rich conditions promote rapid frond propagation, nutrient starvation triggers a profound developmental switch. This stress response leads to the accumulation of starch and lipids and is a key signal for the formation of dormant turions (Pasaribu et al. 2023).

#### Phytohormonal regulation

Environmental signals are often mediated by phytohormones. ABA is a central regulator of stress responses and dormancy. Ethylene, ABA, and salicylic acid jointly orchestrate aerenchyma formation (Kim et al. 2024). Furthermore, exogenous application of ABA under nutrient starvation conditions synergistically enhances starch accumulation, demonstrating how hormonal and environmental pathways intersect (Pasaribu et al. 2023).

#### Ionome and structural evolution

The plant ionome (elemental composition) is tightly linked to its body plan. Comparative analysis across duckweed genera reveals a clear trend: species with reduced structural complexity (e.g., rootless *Wolffia*) show significantly lower tissue calcium and magnesium content compared to the ancestral, rooted *Spirodela*. This "ionomic remodeling" correlates with the loss of structural components like cell walls and roots and influences the plant's metal uptake profile (Smith et al. 2024).

### Developmental switching: the turion life cycle

A quintessential example of duckweed's environmental developmental plasticity is the formation of turions, that is, dense, dormant vegetative propagules that enable survival during winter or drought.

#### Induction and formation

Turion development is induced by adverse conditions such as nutrient shortage, crowding, and shortening days. ABA signaling plays a confirmed key role in initiating this dormancy program. The transformation involves the reprogramming of frond meristems: cell division ceases, cells thicken, and storage compounds (primarily starch, but also lipids) are massively accumulated (Pasaribu et al. 2023).

#### Dormancy and molecular physiology

Mature turions sink and enter a dormant state. Transcriptomic comparisons between fronds and turions highlight the drastic molecular shift, with upregulation of pathways for stress tolerance, starch/lipid metabolism, and dormancy (Pasaribu et al. 2023). Evidence also suggests epigenetic changes, such as cytosine methylation, help lock in this dormant state (Pasaribu et al. 2023).

#### Germination and reactivation

Breaking dormancy requires specific environmental signals, primarily prolonged cold (stratification) followed by warming and light (Ziegler 2024). Germination involves the rapid mobilization of stored starch and lipids to fuel the outgrowth of a new frond, reactivating the vegetative growth cycle (Ziegler 2024). Interestingly, asynchronous reactivation of turions in a population may be a bet-hedging strategy to spread risk in variable environments.

## Decoding the minimalist blueprint: genomic, epigenomic, and systems insights

The advent and application of high-throughput omics technologies have revolutionized duckweed research, transitioning it from a physiological oddity to a well-defined model system. Genomics, transcriptomics, epigenomics, and metabolomics have collectively unraveled the genetic and molecular underpinnings of its extreme biology (Table 1). These studies not only elucidate the unique evolutionary trajectory and cellular adaptations of duckweeds but also provide the essential toolkit for their rational engineering as a sustainable plant chassis for biotechnology.

**Table 1:** Milestones in duckweed omics resources

Resource Type	Species	Key Findings/Contribution	Ref	Data access
Genome	*Spirodela polyrhiza*	High-quality chromosome-scale assembly; insights into gene family contraction and expansion	(An et al. 2019)	SWLF00000000.1
Genome	*Lemna minor*	The first draft genome of *Lemna minor*	(Van Hoeck et al. 2015)	SRP065561—SRA—NCBI
Genome	*Landoltia punctata*	Reveal progressive gene loss from *Spirodela* to *Wolffia*	(Fang et al. 2025)	ID 546087—BioProject—NCBI
Genome	*Wolffiella lingulata*	N/A	N/A	ID 71207—BioProject—NCBI
Genome	*Wolffia australiana*	Help explain its specialized physiology and unique morphology	(Park et al. 2021)	ID 611905—BioProject—NCBI
Epigenome	*Spirodela polyrhiza*	Simplified RdDM pathway; low methylation and alternative chromatin marks	(Harkess et al. 2024)	ID 675910—BioProject—NCBI
Epigenome	*Spirodela polyrhiza*	DNA methylation-independent TE silencing mechanisms	(Dombey et al. 2025)	ID 1164696—BioProject—NCBI
Single-Cell Transcriptome	*Wolffia australiana*	Only four major cell types, highlighting extreme organismal streamlining	(Denyer et al. 2024)	ID 1124135—BioProject—NCBI
Single-Cell Transcriptome	*Lemna minuta*	Cell type atlas and expression of elemental transport genes	(Abramson et al. 2022)	SAMN19243672—SRA—NCBI
Metabolome & GWAS	*Spirodela polyrhiza*	Genetic trade-off between growth and specialized metabolite accumulation	(Hofer et al. 2025)	ID 701543—BioProject—NCBI; ID 934173—BioProject—NCBI; Genome-Wide Association Study of Metabolic Traits in the Duckweed Spirodela polyrhiza—Mendeley Data
Proteome	*Spirodela polyrhiza*	High abundance of photosynthesis and carbohydrate metabolism proteins	(Harkess et al. 2021)	ID 631098—BioProject—NCBI; ID PXD017093—Browse ProteomeXchange Datasets

### Genomic insights into reductive evolution and structural simplification

High-quality, chromosome-scale genome assemblies for key species like *Spirodela polyrhiza* and *Lemna minor* have provided the foundational resource for understanding duckweed biology. The comparison of genomes across the five duckweed genera reveals a clear narrative of reductive evolution, a process of genomic streamlining that mirrors the simplification of their body plan as they adapted to an aquatic, clonal lifestyle (An et al. 2019; Fang et al. 2025; Hoang et al. 2022).

#### Gene family dynamics

Comparative genomics demonstrates a progressive, genus-by-genus contraction of gene families associated with lost or reduced structures and functions. For instance, gene families responsible for lignocellulose biosynthesis, root development, and stomatal function show systematic reductions from the ancestral, rooted *Spirodela* to the derived, rootless *Wolffia* (Fang et al. 2025). Most recently, a chromosome-scale genome assembly of *Wolffia australiana*, the most structurally reduced duckweed, was achieved, revealing a highly complete genome (Benchmarking Universal Single-Copy Orthologs, BUSCO, 94.55%) with a contig N50 of 18.6 Mb (Li et al. 2023). This genome provides molecular evidence for the rootless phenotype, showing substantial contraction of genes involved in auxin signaling and vascular development compared to those in *Arabidopsis* (Li et al. 2023). This genomic erosion underpins the observed loss of structural complexity and supports the "back-to-water" evolutionary model.

#### Specialized expansions

Despite overall reduction, targeted gene family expansions are evident and linked to niche adaptation. *Spirodela polyrhiza* shows significant tandem duplications of disease-resistant genes, including those encoding antimicrobial peptides and dirigent proteins (An et al. 2019). Similarly, an expanded flavonoid biosynthesis pathway is implicated in ultraviolet (UV) protection and antioxidant defense for a floating lifestyle (Fang et al. 2025). These expansions contrast with the general trend of gene loss and highlight evolutionary priorities.

#### Chromosomal and ploidy landscape

Cytogenomic studies have cataloged the chromosome numbers and genome sizes of all 36 duckweed species, revealing a complex history of whole-genome duplications, hybridization, and polyploidy (Ernst et al. 2025; Hoang et al. 2022). Notably, triploid hybrids are common, facilitated by the loss of genetic pathways enforcing reproductive isolation (the "triploid block") (Ernst et al. 2025). This genomic plasticity has implications for breeding and underscores the need for precise genotyping tools, such as polymorphic NB-ARC loci or reduced-representation sequencing (Genotyping-by-Sequencing, GBS), to track clonal lines (Bog et al. 2022).

### Epigenomics and chromatin: unconventional regulation in a clonal world

Duckweeds challenge established paradigms of plant epigenetics. In contrast to model plants like *Arabidopsis*, the clonal, fast-cycling lifestyle of duckweeds is associated with a radically simplified epigenetic landscape.

#### Atypical DNA methylation and siRNA systems

*Spirodela polyrhiza* has lost key components of the canonical RNA-directed DNA methylation (RdDM) pathway, resulting in very low levels of 24-nucleotide small interfering RNAs (siRNAs) and a drastic reduction in cytosine methylation within gene bodies and repetitive elements (An et al. 2019; Harkess et al. 2024). Despite this, its genome remains stable, with transposable elements (TEs) not running rampant.

#### Divergent silencing mechanisms

Research presents a nuanced picture of TE control. While most degenerated TEs lack DNA methylation, they are marked by alternative heterochromatin modifications like H3K9me1 and H3K27me1, suggesting a DNA methylation-independent maintenance system (Dombey et al. 2025). Intact, potentially active TEs are still targeted by a residual, focused RdDM mechanism involving 24 nt siRNAs, DNA methylation, and H3K9me2 (Dombey et al. 2025). This reveals a selective, streamlined silencing strategy adapted for clonality.

### Transcriptomics and single-cell resolution: decoding physiology and cellular simplicity

Transcriptomic studies bridge the gap between genome and phenotype, elucidating dynamic responses to the environment and the functional organization of duckweed's minimalist body.

#### Bulk transcriptomics and environmental responses

RNA-seq analyses have mapped transcriptional networks underlying key traits. Studies on turion dormancy have identified master regulators of starch/lipid metabolism and stress tolerance involved in this developmental switch (Pasaribu et al. 2023; Ziegler 2024). Light quality (red vs. blue) differentially regulates pathways for starch versus protein accumulation, providing a dial to tune biomass composition for end-use (e.g., biofuel vs. feed) (Zhong et al. 2021).

#### Pioneering single-cell transcriptomics

Recent studies have begun applying single-cell/nuclei RNA-seq to duckweeds. In the highly reduced *Wolffia australiana*, this approach resolved just four principal cell clusters (aquatic/aerial parenchyma and epidermis), with surprisingly few genes defining tissue specializations, affirming its status as a "streamlined organism" (Denyer et al. 2024). Complementing single-cell studies in *Wolffia australiana*, Li et al. (2023) generated the first single-nucleus RNA-seq atlas for this species, revealing expression of genes typically associated with tissues absent in the plantlet and providing insights into the relationship between gene content and morphological simplification. A preliminary cell atlas of *Lemna minuta* also defined putative cell types and highlighted mesophyll cells with high expression of elemental transport genes, correlating with phytoremediation capacity (Abramson et al. 2022). These studies represent the first steps toward a full cellular map of an entire plant.

### Metabolomics and integrative omics: linking genotype to phenotype

Connecting the genome to the metabolome is critical for engineering metabolic output. Metabolomics and genome-wide association studies (GWAS) are now uncovering the genetic architecture of duckweed's chemical composition.

#### Metabolic GWAS

A landmark GWAS on 137 *Spirodela polyrhiza* genotypes analyzed 42 metabolites, revealing a fundamental growth-metabolism trade-off: biomass correlated positively with free amino acids (e.g., glutamine) but negatively with specialized metabolites like flavonoids (Hofer et al. 2025). This identifies a key engineering challenge: boosting valuable compounds without crippling growth. This growth-metabolism trade-off has direct and actionable implications for engineering duckweed as a chassis. First, it establishes a quantitative framework for metabolic engineering: any strategy aimed at enhancing the accumulation of high-value specialized metabolites (e.g., flavonoids, terpenoids, or pharmaceutical precursors) must anticipate potential penalties in biomass productivity. The GWAS data provide a rational basis for selecting engineering targets by identifying genetic nodes that govern this balance. For example, genes associated with photosynthesis and protein degradation emerge as high-priority targets because manipulating them could potentially reallocate carbon and nitrogen fluxes between primary and secondary metabolism. Second, the trade-off informs the design of inducible production systems. Rather than constitutively expressing pathways for specialized metabolites, which would chronically suppress growth, engineers can leverage the negative correlation to design two-phase cultivation strategies. In this approach, a first phase optimizes conditions for rapid biomass accumulation (e.g., nutrient-rich media, optimal light). Upon reaching desired biomass, a second phase is triggered by environmental or chemical inducers that activate expression of the target metabolic pathway, temporarily diverting resources from growth to product accumulation. This "growth then production" strategy, widely used in microbial fermentation, is now genetically grounded for duckweed by the GWAS data.

#### Candidate genes for engineering

The same study pinpointed candidate genes jointly associated with multiple metabolic traits, including those involved in photosynthesis, protein degradation, and organ development (Hofer et al. 2025). These are prime targets for multiplexed editing to optimize both yield and metabolic content simultaneously. These candidate genes identified offer precise entry points for genome editing. Rather than broad, untargeted approaches, metabolic engineers can now design CRISPR-Cas9 strategies to: (i) knockout negative regulators of specialized metabolism, (ii) introduce promoter variants that uncouple growth and production, or (iii) edit transcription factor binding sites to fine-tune expression of pathway genes. For instance, editing a single gene involved in protein degradation could simultaneously influence nitrogen remobilization and carbon partitioning, potentially increasing both biomass and target metabolite yields in optimized backgrounds.

Finally, these findings refine the selection of appropriate duckweed genotypes for specific applications. The GWAS reveals natural variation in the growth-metabolism balance across accessions. For industrial production of high-value metabolites, researchers can now screen for and select genotypes with inherently favorable trade-off ratios, those that achieve higher specialized metabolite content with minimal biomass penalty, providing an elite starting material for further engineering. Conversely, for applications prioritizing maximal biomass (e.g., animal feed or starch production), genotypes at the opposite end of the spectrum can be chosen.

In summary, the GWAS-derived growth-metabolism trade-off transforms metabolic engineering in duckweed from a trial-and-error endeavor into a data-driven discipline. It provides both a conceptual framework and a molecular roadmap for designing strains, cultivation strategies, and genetic interventions that optimally balance productivity and yield.

## Building the toolbox: from foundational transformation to precision genome engineering

**Fig. 2:**
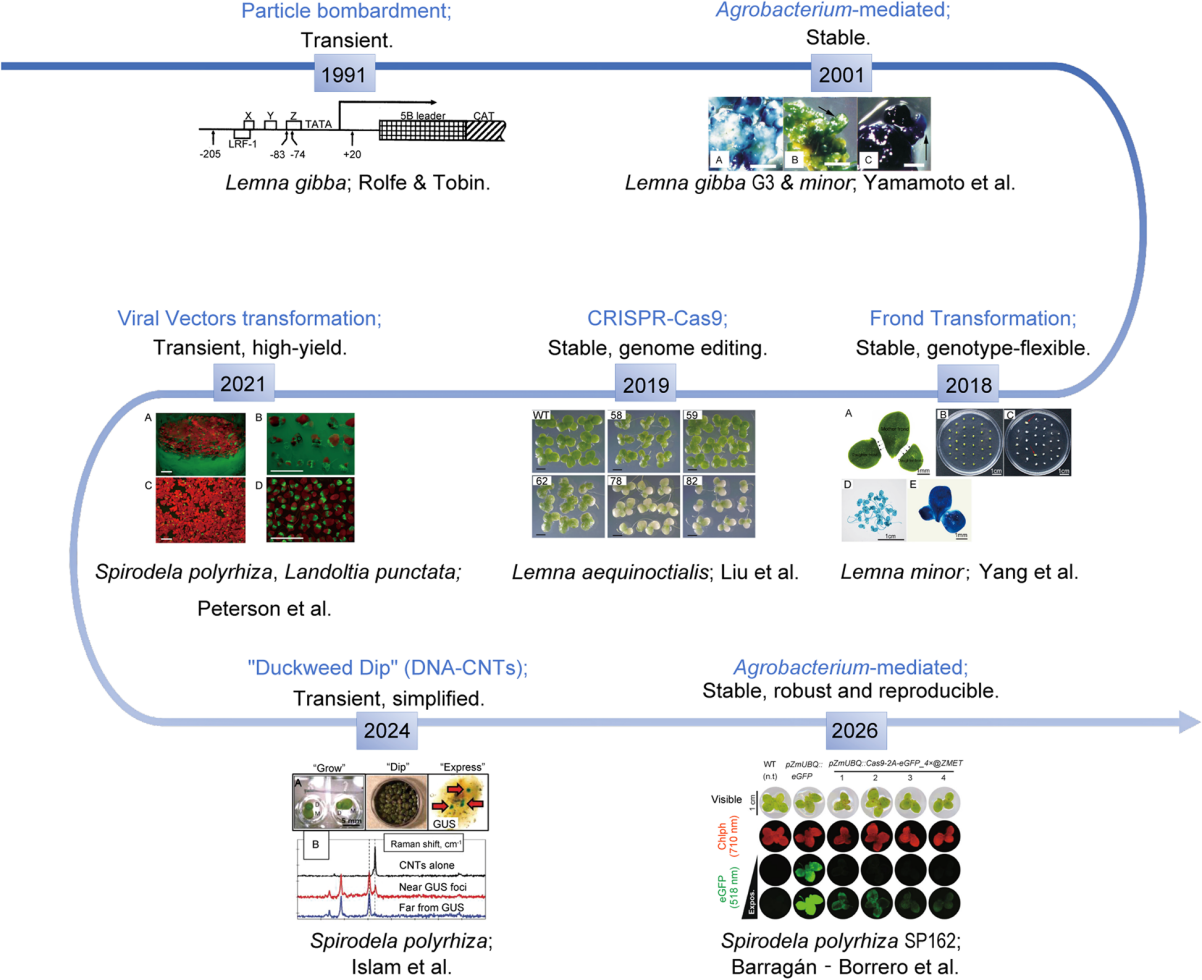
A timeline of genetic transformation advances for duckweeds. Major milestones are plotted against a timeline (approximate years), showing the improvements of transformation methods. Milestones include the first stable genetic transformation via callus, the development of more efficient frond transformation methods, the simple “duckweed dip” protocol, the implementation of CRISPR-Cas9 for precise gene editing, the high-yield or simplified transient transformation and the robust and reproducible system. Inset graphs conceptually illustrate the trend of increasing efficiency or decreasing time-to-transgenic plant generation facilitated by these advances. All schematic illustrations are derived from corresponding key references, representing each significant methodological advance or technical workflow

The journey to genetically manipulate duckweeds mirrors the broader story of establishing a new model system: a path from challenging and time-consuming initial successes to increasingly streamlined, efficient, and versatile tools (Fig. 2, Table 2). This section reviews the historical progression of these technologies, emphasizing quantitative metrics, namely efficiencies, timelines, species/strain dependencies, etc., to provide readers with actionable guidance on “what works, when, and why”. Table 2 summarizes key parameters from major transformation studies, enabling direct comparison of methods and outcomes.

**Table 2:** Duckweed transformation: from foundational transformation to precision genome engineering

Year	Method/System	Species Tested	Max Efficiency (%)	Time frame (weeks)	Key Boundary Condition	Reported advantage	Note
1991 (Rolfe And Tobin 1991)	Particle bombardment	*Lemna gibba*	Not reported	transient	Etiolated tissue, dark pre-culture, 1.2 μm tungsten particles	Rapid promoter analysis; phytochrome-responsive elements mapped	Best for functional promoter testing; not for line generation
2001 (Boehm et al. 2001)	*Agrobacterium*-mediated transient transformation	W*olffia crassum*	3.9—30%	transient	Vir gene induction; require wounding	First transient system in *Wolffia*; vacuum targets meristem	Use vacuum for meristem targeting; corundum for epidermal cells
2001 (Yamamoto et al. 2001)	*Agrobacterium*-mediated nodule transformation	*Lemna gibba G3* *L.minor *8627/8744	Not reported	3—6	Optimized nodule induction media	Stable, heritable transformation	Reliable for stable line generation in *Lemna* spp.
2007 (Vunsh et al. 2007)	*Agrobacterium*-mediated callus transformation	S*pirodela oligorrhiza*	> 25%	8—12	Callus induction; high-expression vectors	Recombinant soluble protein yield > 25%	Best for high-value protein production
2011 (Chhabra et al. 2011)	*Agrobacterium*-mediated nodular callus	*Lemna minor*	3.8%	11—13	Specific ecotype; nodular callus induction	First report for Indian isolate	Use only if isolate-specific traits needed
2018 (Yang et al. 2018)	Frond transformation system (FTS)	*Lemna minor*	> 50%	4—6	Direct frond infection; genotype-flexible medium	Fast; work across multiple genotypes	Best for rapid stable transformation screening
2019 (Liu et al. 2019)	CRISPR-Cas9-based genome editing	*Lemna aequinoctialis*	76.2%	8—12	*Agrobacterium*-mediated; sgRNA design critical	First stable genome editing in duckweed	Use for functional gene knockout; require validation
2021 (Peterson et al. 2021)	Transient expression (viral vectors)	*Spirodela polyrhiza*, *Landoltia punctata*	1.24 ± 0.13 mg/g FW	Transient	Viral vector; agro-infiltration	Rapid protein production; high yield	Ideal for rapid protein screening, no stable lines
2024 (Islam et al. 2024)	Floral dipping	*Spirodela polyrhiza*	100%	Transient	Carbon nanotube delivery	Simplified delivery; no need for sterility	Promising for field application; need validation
2025 (Islam et al. 2025)	High-efficiency platform	*Spirodela polyrhiza*	> 90%	Transient/stable (12–16 weeks)	Vacuum infiltration; two-step regeneration (TDZ/zeatin → liquid SH medium)	High transformation efficiency; stable transgene expression > 270 days	Future tool
2026 (Barragán‐Borrero et al. 2026)	Robust-Reproducible platform	*Spirodela polyrhiza SP162*	Not yet reported	Transient/stable (12–16 weeks)	use of *pZmUBQ* promoters; two-step regeneration (TDZ/zeatin → liquid SH medium)	Genome browser and gRNA design tool available; stable transgene expression over 6 months	Future tool; monitor literature

### Foundational work and the era of callus-based transformation

The successful genetic transformation of duckweed in the late 20th and early 21st century was a critical proof-of-concept, though methods were complex and species-dependent. Initial reports, primarily in *Lemna gibba* and *Lemna minor*, established *Agrobacterium tumefaciens*-mediated transformation as the primary method (Yamamoto et al. 2001). This work formed the backbone of subsequent protocol development. A significant milestone was reached when soluble recombinant GFP protein was purified in stably transformed *Spirodela polyrhiza* plants (Vunsh et al. 2007), suggesting duckweed as a potential bio-factory for target production. A landmark study by Chhabra et al. (2011) with an Indian isolate of *Lemna minor* exemplified the intricate process of early transformation. It involved inducing hard nodular calli from daughter fronds using specific combinations of auxins and cytokinins, co-cultivating with *Agrobacterium*, and then regenerating transgenic plants over 11–13 weeks, achieving a transformation frequency of 3.8% (Chhabra et al. 2011). The dependence on specific plant growth regulators like BAP, 2,4-D, and TDZ, co-culture conditions, and selection agents was clearly established. Similar callus-dependent protocols were developed for other species, including *Wolffia arrhiza*, which required unique two-step hormonal treatments for callus induction and maintenance (Khvatkov et al. 2015). These early protocols confirmed that stable, heritable transgene integration was possible but highlighted major bottlenecks: long timelines (often several months), low efficiency, and significant genotype constraints, as many elite or wild strains proved recalcitrant to callus induction and regeneration.

### Protocol optimization and emergence of direct frond transformation

The 2010s saw concerted efforts to refine protocols and reduce dependencies on difficult tissue culture steps. Research focused on optimizing factors known to affect *Agrobacterium* virulence and plant cell competence, such as the concentration of acetosyringone, co-culture duration, bacterial strain, and explant type. For instance, studies on *Lemna aequinoctialis* identified that a co-cultivation medium with 200 µM acetosyringone for one day in the dark was crucial, improving transformation efficiency (Wang et al. 2021). The most significant advancement in stable transformation during this period was the development of the Frond Transformation System (FTS) (Yang et al. 2018). This paradigm-shifting approach bypassed the lengthy callus phase entirely. Instead, *Agrobacterium* was used to directly infect intact fronds or frond pieces, from which transgenic plants could regenerate (Yang et al. 2018). Compared to the Conventional Callus Transformation System (CTS) requiring 8–9 months, FTS produced stable transgenic lines in approximately 3 months and, crucially, was applicable to a wider range of *Lemna minor* genotypes that were previously non-transformable due to poor callus induction (Yang et al. 2018). This work demonstrated that efficient, genotype-flexible stable transformation was achievable.

### Expansion of toolkits: transient expression and endogenous promoters

Parallel to improvements in stable transformation, researchers developed powerful transient expression systems. As demonstrated by Peterson et al. (2021), using deconstructed viral vectors (e.g., from *Potato virus X*) in *Spirodela polyrhiza* and in *Landoltia punctata* enabled extremely high-yield protein production (> 1 mg/g fresh weight) within days (Peterson et al. 2021). These systems are ideal for rapid protein production, functional gene screening, and bioprospecting without genomic integration. Another critical advancement was moving beyond reliance on the constitutive *35S* promoter from Cauliflower Mosaic Virus. The discovery and characterization of strong, endogenous promoters like *LpSUT2* from *Landoltia punctata* addressed a key limitation: transgene silencing under stress (Wei et al. 2024). Unlike the *35S* promoter, which became methylated and silenced under high antibiotic selection pressure, *LpSUT2* maintained high activity, ensuring robust and stable expression of heterologous proteins, a vital feature for industrial bioproduction.

### The CRISPR-Cas9 revolution and novel delivery methods

The adaptation of CRISPR-Cas9 genome editing to duckweeds, first reported by Liu et al. (2019) in *L. aequinoctialis* (Liu et al. 2019), marked the transition from transgenics to precise genome engineering. By optimizing their *Agrobacterium*-mediated transformation protocol to a rapid 5–6 week cycle with > 94% success, they achieved a 14.3% success rate in generating biallelic mutants. This opened the door to targeted gene knockouts for functional genomics and trait engineering. The most recent frontier is the development of radically simplified, "hands-off" delivery methods designed for high-throughput synthetic biology. The "duckweed dip" method (Islam et al. 2024) represents this trend. It involves simply incubating *Spirodela polyrhiza* in a solution containing plasmid DNA wrapped around carbon nanotubes (DNA-CNTs), which the plants take up directly from the medium to express transgenes. This method eliminates the need for *Agrobacterium* or complex physical infiltration. Building on this, Islam et al. (2025) reported a fully optimized, high-efficiency platform for *Spirodela polyrhiza* achieving > 90–100% efficiency at each stage, from callus induction to stable transformation and visual marker-free selection, with the entire process taking weeks instead of months (Islam et al. 2025). At the same time, Barragán-Borrero et al. (2026) established a robust and reproducible transformation system using *Spirodela polyrhiza SP162*, which has not only publically available genome information but also online tools for genome editing (Barragán-Borrero et al. 2026). These innovations aim to dismantle the final major bottlenecks to routine genetic manipulation.

### Reproducibility and protocol standardization for duckweed transformation

A critical yet underappreciated challenge in the field is the reproducibility of transformation protocols across laboratories and even across independent experiments within the same laboratory (Table 2). The literature contains conflicting reports on transformation efficiencies, with some studies claiming near-perfect efficiencies (> 90%) while others report far lower success rates under ostensibly similar conditions (Table 2). These discrepancies likely arise from multiple factors: undocumented variations in plant physiological state, differences in *Agrobacterium* strain preparation, subtle differences in tissue culture conditions, and lack of standardized metrics for reporting efficiency. As recently articulated in the literature, reproducibility remains a central bottleneck limiting the broader adoption of duckweed as a mainstream genetic system.

To address these challenges, the community should consider the following priorities: (i) Development of reference materials and positive controls. Establishing universally accessible reference lines (e.g., a well-characterized *Spirodela polyrhiza* line with stable GFP expression) would enable laboratories to validate their protocols before attempting new transformations. (ii) Standardized reporting guidelines. The field would benefit from consensus metrics for reporting transformation efficiency, distinguishing between transient expression, stable integration, and germline transmission, as well as mandatory documentation of critical parameters (e.g., plant growth conditions prior to transformation, bacterial density, co-culture duration and temperature, selection regime). Table 2 in this review represents a first step toward compiling such comparative data. (iii) Independent validation studies. Key "high-efficiency" protocols should be subjected to independent validation by multiple laboratories, with results published regardless of outcome, to establish genuine robustness across settings. (iv) Mechanistic understanding of recalcitrance. Rather than treating transformation as a black box, systematic investigation of why certain genotypes or physiological states resist transformation could reveal fundamental biological barriers and inform rational protocol design.

In summary, while the trajectory of duckweed genetic tool development has been impressive, the field must now mature by confronting reproducibility challenges head-on. The next generation of advances will depend not only on higher reported efficiencies but on protocols that are demonstrably robust, transferable, and validated across the diverse species and strains that comprise the *Lemnaceae* family.

## Duckweed in sustainable agriculture and environmental remediation

**Fig. 3:**
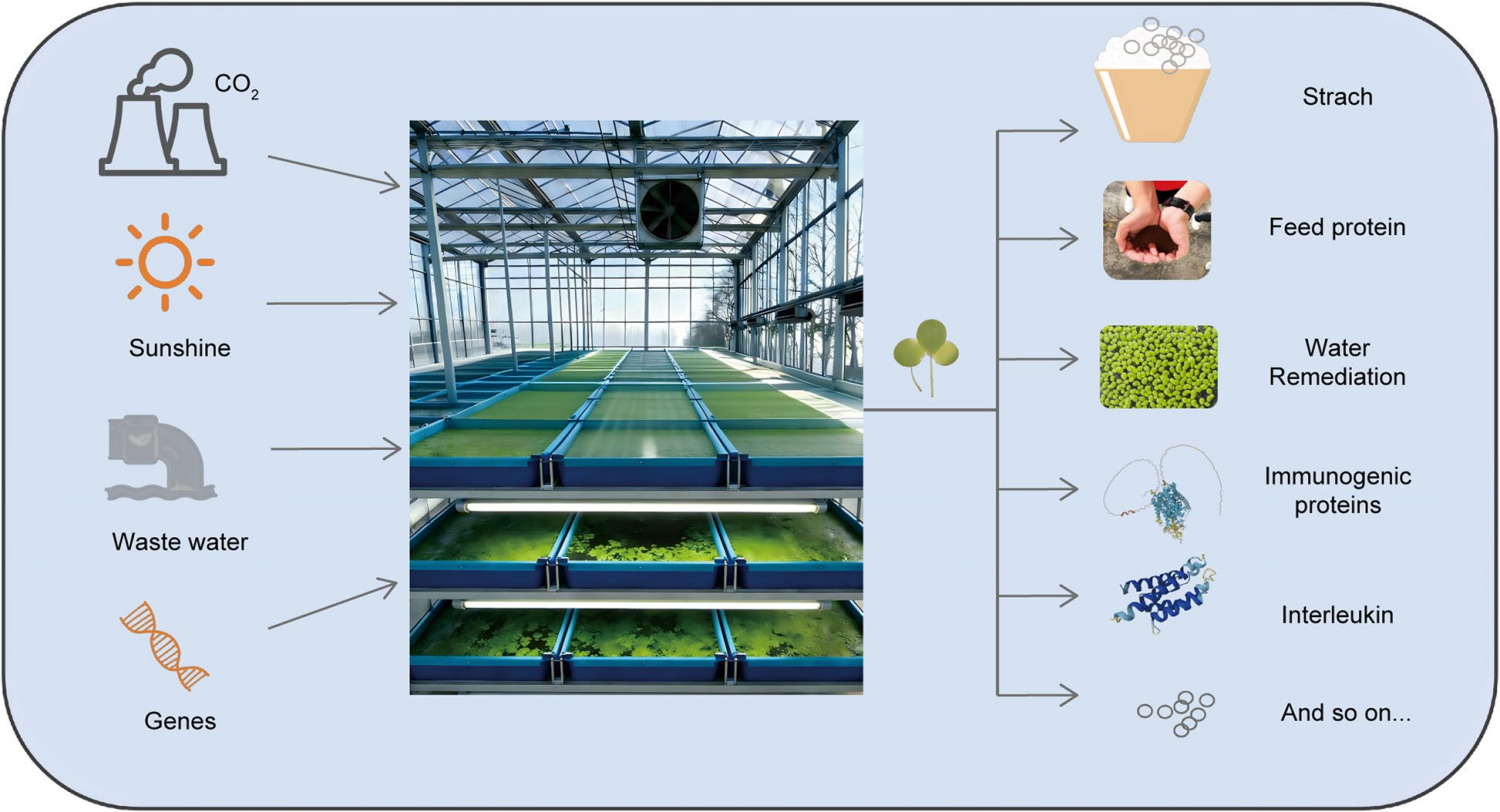
Duckweeds as integrated biotech platforms. A duckweed frond "factory". The duckweed fronds work as a central, sustainable “bio-factory.” Arrows indicate the flow of low-cost or waste inputs (light, atmospheric CO₂, wastewater, and engineered genetic constructs) into the system. The biotech platform (duckweed) converts these inputs into target bioproducts as outputs, including starch, feed, ecosystem restoration, recombinant vaccines, therapeutic proteins like interleukins and so on

While duckweed is now celebrated as a promising chassis for synthetic biology, its practical value to human society is deeply rooted in history (Fig. 3). For centuries, these fast-growing aquatic plants have been utilized in traditional agriculture and environmental management across Asia and other regions, valued as a nutritious supplement for livestock and fish, a natural purifier of nutrient-rich water, and a reliable source of biomass (Coughlan et al. 2022; Sembada et al. 2024). Contemporary research is now rigorously validating these traditional uses with modern scientific methods, quantifying their efficacy and uncovering the sophisticated biological mechanisms that make duckweed exceptionally effective for these roles. This body of work reinforces duckweed's foundational utility and provides a robust springboard for its advanced biotechnological applications.

The use of duckweed as a high-protein animal feed is one of its most established applications, consistently supported by modern zootechnical studies. Recent research confirms that duckweed can be successfully incorporated into poultry diets without compromising productivity (Baghban-Kanani et al. 2023). For instance, replacing significant portions (up to 15%) of conventional ingredients like wheat and soybean meal with *Lemna minor* in the diets of laying hens did not affect egg production or weight (Baghban-Kanani et al. 2023). It positively enhanced yolk color, likely due to increased xanthophylls, and showed indications of hepatoprotective effects, as evidenced by reduced serum liver enzyme levels (Baghban-Kanani et al. 2023). This demonstrates that duckweed is not merely a filler but a functional feed component that can contribute to animal health and product quality while improving land-use efficiency by providing an alternative protein source that does not compete with staple crops. The nutritional composition of duckweed biomass can be deliberately manipulated through cultivation conditions. A systematic study of nitrogen and phosphorus availability in *Lemna minor* demonstrated that combined nitrogen and phosphorus application at 30 ppm maximized biomass production (172 g/m^2^/day), while protein content reached 33%, lipid content 10%, and carbohydrates 60% under optimized nutrient regimes (Ullah et al. 2022). Mineral accumulation of Ca, Mg, Fe, Mn, and Zn was also significantly affected by medium composition, providing a framework for producing nutritionally tailored biomass for specific feed applications.

In environmental management, duckweed's proficiency in phytoremediation is being precisely dissected. Its dense mat-forming growth provides a multifunctional, nature-based solution for improving water quality and mitigating agricultural pollution (Zhou et al. 2023). A compelling example is its role in mitigating ammonia (NH₃) volatilization, a major nitrogen loss pathway and source of air pollution from irrigated paddies (Liu et al. 2024). Field studies have shown that a complete duckweed cover on paddies can almost entirely offset the increase in ammonia emissions induced by water-saving irrigation techniques like alternate wetting and drying (Liu et al. 2024). The mechanism is tripartite: the physical mat blocks gas exchange (accounting for ~ 51% of the effect), the plants directly absorb ammonium from the water (~ 28%), and the cover reduces water temperature to further inhibit volatilization (~ 21%) (Liu et al. 2024). This positions duckweed as a powerful tool for sustainable agricultural intensification, allowing for water conservation without the environmental penalty of increased air pollution. Furthermore, duckweed's interactions with emerging contaminants like nanoplastics are being elucidated at unprecedented resolution. Single-cell transcriptomic studies reveal that duckweed exhibits cell-type-specific stress responses to nanoplastics, with mesophyll and epidermal cells activating distinct detoxification and metabolic pathways (Yuan et al. 2025). Intriguingly, a significant portion (36.8—51.4%) of absorbed nanoplastics can be excreted upon transfer to clean water, demonstrating an active, dynamic response rather than passive accumulation (Yuan et al. 2025). This deep mechanistic insight into its stress response and recovery potential underscores duckweed's inherent resilience and refines its profile as a sensitive bio-indicator and remediator in polluted ecosystems.

Perhaps the most dramatic modern validation of a traditional use is in starch production. Long recognized for accumulating starch, duckweed's potential is now being hyper-accelerated through optimized cultivation strategies. By combining nutrient limitation, a classic stressor that triggers carbon partitioning toward starch, with elevated CO₂ supplementation, researchers have achieved remarkable yields. In *Landoltia punctata*, this dual approach resulted in a starch content reaching over 72% of dry weight and a productivity of 10.4 g per square meter per day (Guo et al. 2025). Integrated multi-omics analyses reveal the orchestrated molecular shift behind this hyper-accumulation: key enzymes in the starch biosynthesis pathway are significantly upregulated, while pathways for competing products like lignocellulose and proteins are downregulated (Guo et al. 2025). This efficient channeling of photosynthetic carbon, achievable in simple aquatic systems without arable land, establishes duckweed as a compelling and highly productive non-traditional crop for industrial starch, with clear implications for biofuel and biopolymer industries.

Collectively, this research on feed, remediation, and starch production does more than confirm historical practices; it provides a critical performance baseline and a mechanistic understanding of duckweed's innate capabilities. These proven applications form the essential real-world foundation upon which more advanced genetic and bioprocessing engineering, explored in the following section, can be securely built to develop next-generation duckweed biotechnologies.

## Duckweed as a next-generation chassis: engineering a sustainable biofactory

The emerging field of plant molecular farming seeks to transform plants into efficient, scalable, and sustainable bioreactors. While traditional systems like tobacco (*Nicotiana benthamiana*) have pioneered this space, duckweed is rapidly gaining prominence as a uniquely advantageous next-generation chassis (Table 3). This section reviews the progress in engineering duckweeds for bioproduction (Fig. 3), contextualizes these advances by comparing duckweed to other established and emerging plant chassis, and outlines its specific competitive advantages for industrial translation.

**Table 3:** Comparative analysis of plant-based bioproduction chassis

Feature	Duckweed	*Nicotiana benthamiana*	Microalgae
Growth Rate	Doubling in 1—3 days (Stomp 2005)	Weeks to harvest (Song et al. 2024)	Species-dependent (Rodolfi et al. 2009)
Cultivation System	Low cost, aquatic, soil-free, contained (Xu et al. 2011)	Soil/hydroponic (Nguyen et al. 2023)	High cost, aquatic, soil-free, contained (Wijffels And Barbosa 2010)
Genetic Tools	CRISPR, stable/transient transformation (Chhabra et al. 2011;Islam et al. 2024; Islam et al. 2025; Liu et al. 2019; Peterson et al. 2021; Vunsh et al. 2007;Yamamoto et al. 2001; Yang et al. 2018)	CRISPR, transient & stable systems (Garcia-Perez et al. 2025; Goodin et al. 2008; Norkunas et al. 2018; Vollheyde et al. 2023)	Species-dependent, efficient in some (León and Fernández 2007)
Biosafety	High (aquatic, few pollen dispersal) (Fourounjian et al. 2021, Lee et al. 2025)	Medium (field cultivation risks) (Wilkinson et al. 2003)	High (contained cultivation) (Wijffels And Barbosa 2010)
Downstream Processing	Simplified cell walls, easier extraction (Sowinski et al. 2019; Zhao et al. 2014)	Lignin-rich, complex extraction (Le Mauff et al. 2017; Vollheyde et al. 2023)	Variable cell walls, cost-dependent (Abdullah et al. 2024)
Edibility/Safety	High (traditional food/feed) (Baek et al. 2021)	Low (alkaloids, secondary metabolites) (Shen et al. 2024; Vollheyde et al. 2023)	Variable (Kusmayadi et al. 2021)
Typical Products	Vaccines, oral proteins, feed additives (Firsov et al. 2015; Ko et al. 2011; Tan et al. 2022, 2025)	Recombinant proteins, antibodies, vaccines (Song et al. 2024)	Oils, pigments, recombinant proteins (Abdullah et al. 2024; Gimpel et al. 2013; Gong et al. 2011; Kusmayadi et al. 2021; Patel et al. 2022)
Scalability	High (vertical farming) (von Salzen et al. 2025)	Medium (requires land/facilities) (Wilkinson et al. 2003)	High (industrial photobioreactors) (Kirnev et al. 2020; Wijffels And Barbosa 2010)

The foundational work establishing duckweed as a viable expression platform has focused primarily on producing antigens and immunomodulators for veterinary and medical applications, demonstrating both stable transformation and functional efficacy of the recombinant products. The first report of an animal vaccine antigen expressed in duckweed was for the Porcine Epidemic Diarrhea Virus (PEDV) spike protein in *Lemna minor*, confirming the feasibility of producing immunogenic proteins in this system (Ko et al. 2011). This was followed by the high-yield expression (up to 1.96% of total soluble protein) of the conserved M2e peptide from avian influenza virus H5N1 in duckweed, highlighting its potential for producing "universal" vaccine components (Firsov et al. 2015). More recently, a highly efficient transformation protocol (exceeding 85% efficiency) was developed in *Lemna minor* and used to produce chicken interleukin-17B (chIL-17B) (Tan et al. 2022) and the EpiC vaccine (Tan et al. 2025). This cytokine, when administered orally in transgenic duckweed biomass, functioned as an effective mucosal vaccine adjuvant, boosting both systemic and mucosal immunity in chickens. These studies collectively validate duckweed's capacity to produce biologically active, complex proteins that retain their function in downstream applications.

### The strategic advantages of duckweed as a production platform

The comparison underscores a set of convergent advantages that position duckweed uniquely for commercial biomanufacturing, particularly for applications involving direct consumption or where containment is paramount.

#### Unmatched productivity and efficiency

Duckweed's most compelling attribute is its exponential growth rate, among the fastest of any vascular plant (Acosta et al. 2021; Stomp 2005). This translates to potentially shorter production cycles and higher volumetric yields of recombinant products per unit time and infrastructure. Its growth in simple mineral nutrient solutions eliminates dependence on arable land or soil, enabling vertical farming in controlled environments.

#### Inherent safety and "biological containment"

When cultivated in contained, indoor systems, such as bioreactors or controlled-environment vertical farms, duckweed offers significant biosafety advantages. As an obligate aquatic plant with highly reduced roots, its ability to establish and persist in terrestrial ecosystems is extremely limited. Furthermore, its clonal propagation and rare flowering in contained settings minimize the risk of transgene flow via pollen or seed dispersal. However, these biosafety features are contingent upon the implementation of appropriate physical containment measures (e.g., barriers to prevent biomass escape, treatment of wastewater effluent) and do not eliminate the need for location-specific risk assessment, particularly for open-pond cultivation scenarios. This built-in containment potential, when combined with robust cultivation infrastructure, addresses a major regulatory and public concern associated with field-grown transgenic crops. Importantly, unlike tobacco, which produces a suite of biologically active alkaloids and defense compounds that can complicate downstream processing and limit direct use (Wu et al. 2025), duckweed is naturally edible and nutritious, making the raw biomass suitable for direct oral delivery or as a feed additive without extensive purification or detoxification.

#### Downstream processing benefits

The evolutionary reduction of duckweed's body plan extends to its cell wall, which is notably low in lignin and cellulose compared to terrestrial plants (Sowinski et al. 2019; Zhao et al. 2014). This feature can significantly reduce the cost and complexity of downstream processing, whether the goal is to extract intracellular proteins or to digest the biomass for nutrient release.

### Industrial production: current limitations and challenges

Despite its compelling advantages, the industrial-scale production of duckweed as a biomanufacturing platform faces several significant challenges that must be addressed to enable commercial translation.

#### Scalability and cultivation engineering

While laboratory-scale cultivation is well-established, maintaining optimal growth conditions at industrial scale presents formidable engineering hurdles. Duckweed requires precise control of light intensity and quality, temperature, nutrient composition, and pH to achieve its theoretical maximum productivity. In large-scale raceway ponds or bioreactors, light penetration becomes limiting due to self-shading by dense mats, reducing photosynthetic efficiency and biomass yield. Furthermore, achieving and maintaining monodisperse (single-frond) growth, which maximizes light exposure and nutrient uptake, is challenging at scale, as duckweed naturally forms multilayer mats. Developing engineered cultivation systems (e.g., thin-layer cascades, floating photobioreactors) that prevent mat formation while remaining economically viable is a critical engineering priority.

#### Contamination and biosecurity

The open-water nature of duckweed cultivation makes it vulnerable to contamination by algae, bacteria, fungi, and competing duckweed genotypes. Such contaminants can reduce yield, compromise product purity, and outcompete the engineered strain. For pharmaceutical production, maintaining axenic (sterile) conditions is mandatory but becomes exponentially more difficult and expensive at scale. Current sterilization methods (e.g., chemical treatment, UV irradiation) are often impractical for large volumes or may harm the plants. Developing robust, cost-effective strategies for maintaining monoculture integrity in open or semi-closed systems is essential.

#### Genetic and phenotypic stability

The clonal propagation that gives duckweed its rapid growth advantage also poses risks for long-term industrial use. Over successive generations of vegetative reproduction, engineered traits may be lost due to epigenetic silencing, somaclonal variation, or accumulation of spontaneous mutations. While duckweed's simplified epigenome may reduce silencing risks (Dombey et al. 2025; Harkess et al. 2024), empirical data on transgene stability over hundreds of generations under industrial conditions are lacking. Rigorous line selection and periodic quality control are necessary, but these add cost and complexity to manufacturing workflows.

#### Harvesting and downstream processing

Although duckweed's low lignin content facilitates processing, efficient harvesting from large volumes of water remains energetically and economically challenging. Current methods, filtration, centrifugation, or flocculation, are energy-intensive and may damage fronds, leading to loss of intracellular products. For applications requiring purified recombinant proteins, the presence of high levels of native phenolics, oxalates, and proteases in duckweed tissues can interfere with downstream purification and reduce yields. Species- and tissue-specific optimization of extraction and purification protocols is required.

#### Regulatory and public acceptance pathways

As a relatively new platform for pharmaceutical and industrial production, duckweed lacks the established regulatory precedent of microbial systems or well-studied crops like tobacco. For products intended for human or animal use, regulatory agencies will require extensive characterization of the host organism, transgene insertion sites, product consistency, and environmental safety. The "generally recognized as safe" (GRAS) status of duckweed as animal feed provides a foundation, but regulatory pathways for novel recombinant products must be navigated on a case-by-case basis. Public perception of genetically modified aquatic plants for food or pharmaceutical applications also remains an open question.

#### Economic competitiveness

Ultimately, duckweed-based production must compete economically with established systems (microbial fermentation, mammalian cell culture, other plant platforms). While duckweed's low input costs and rapid growth are advantageous, the capital costs of contained cultivation infrastructure, combined with the challenges listed above, may offset these benefits for some products. Detailed techno-economic modeling for specific product classes is needed to identify niche applications where duckweed's unique advantages, particularly edibility and containment, provide clear economic value.

In summary, while duckweed offers transformative potential, realizing this potential requires concerted efforts to address engineering, biological, and regulatory challenges. Many of these limitations are not unique to duckweed but are amplified by its aquatic lifestyle. Progress in scalable bioreactor design, genetic stability testing, and regulatory science will be essential to transition duckweed from a promising chassis to an established industrial workhorse.

## Conclusion and perspectives

The biotechnological promise of duckweed arises from a convergence of unique biological traits that collectively create a superior production platform. Its extreme growth rate, with frond doubling times of 1–3 days, enables short production cycles and exceptional volumetric productivity unmatched among vascular plants. The simplified body plan, characterized by reduced roots, minimal vasculature, and thin fronds, facilitates efficient nutrient uptake, easy harvesting, and a cell wall with low lignin content that simplifies downstream processing. Aquatic, soil-free cultivation allows for vertical farming with precise environmental control, eliminates competition for arable land, and provides built-in biological containment that mitigates transgene escape risks. A streamlined genome, shaped by reductive evolution, offers a simpler genetic background for engineering with fewer redundant pathways. Perhaps most significantly, duckweed possesses a simplified epigenome with reduced DNA methylation and diminished gene silencing pathways, lowering the risk of transgene silencing and enabling more predictable and stable expression of engineered traits (Dombey et al. 2025; Harkess et al. 2024). Its intrinsic edibility and high nutritional value enable direct use as animal feed and create opportunities for "biomass-in-the-product" formats, including oral delivery of biopharmaceuticals. Finally, duckweed's native metabolic capacity for accumulating starch, flavonoids, and other valuable compounds provides a foundation for metabolic engineering that can be tuned via environmental triggers. Together, these traits position duckweed not merely as an alternative to established systems like tobacco or microalgae, but as a strategically distinct platform ideally suited for scalable, contained, and sustainable biomanufacturing.

Duckweed (*Lemnaceae*) has transcended its historical role in traditional agriculture and phytoremediation to emerge as a premier next-generation plant chassis for synthetic biology and sustainable biotechnology. This review has synthesized how its unparalleled combination of biological traits, extreme growth rates, a simplified body plan adapted to contained aquatic culture, intrinsic nutritional value, and a unique epigenetic landscape, converges to create a uniquely advantageous platform. While established systems like tobacco excel in transient protein yield and microalgae offer streamlined genetics, duckweed occupies a strategic middle ground, balancing rapid biomass production, biosafety, and edibility in a way that is ideally suited for scalable, contained biomanufacturing.

The foundational work is firmly in place. High-quality genomic resources for key species like *Spirodela polyrhiza* and *Lemna minor* have illuminated a history of reductive evolution, where the loss of terrestrial complexity (e.g., roots, extensive vasculature) is mirrored by a streamlined genome (Fang et al. 2025; Harkess et al. 2024). This evolutionary trajectory has yielded unexpected biotech advantages, including a simplified epigenome with reduced silencing pathways that may lower barriers to stable transgene expression (Dombey et al. 2025; Ernst et al. 2025). Concurrently, transformation technologies have evolved from lengthy, genotype-dependent callus-based methods to rapid, high-efficiency protocols like the "duckweed dip" (Islam et al. 2024) and optimized *Agrobacterium*-mediated robust and reproducible system (Barragán—Borrero et al. 2026). These advances have been leveraged to produce functional biopharmaceuticals, from vaccine antigens to immune adjuvants, validating duckweed's practical utility (Ko et al. 2011; Tan et al. 2022, 2025).

To transition duckweed from a promising model to an industrial workhorse, future efforts must be directed toward integrated tool development, scalable process engineering, and the exploration of novel product niches. The following interconnected priorities outline the pathway forward:

### Building a unified systems biology platform

The current expansion of omics data (genomics, single-cell transcriptomics, epigenomics, metabolomics) presents a critical bottleneck: integration. A centralized, community-driven data portal, akin to ePlant for *Arabidopsis* (Shiu et al. 2011), is urgently needed. This platform would seamlessly connect a gene locus to its expression patterns across tissues, its epigenetic state, and its influence on metabolic phenotypes. Such a resource is the cornerstone for predictive engineering, allowing researchers to identify optimal promoters, predict epigenetic silencing risks, and select genetic markers for breeding, thereby moving from trial-and-error to rational design.

### Advancing high-throughput genetic toolkits

While transformation is now efficient, the goal is universality and throughput. Priorities include: Universal Protocols: Developing delivery methods (e.g., refined nanoparticle or viral vector systems) that minimize genotype dependence, ensuring all biotechnologically relevant strains are transformable. Synthetic Biology Toolboxes: Creating and characterizing modular, standardized genetic parts libraries, including species-specific promoters (e.g., the stable *LpSUT2* promoter (Wei et al. 2024, terminators, and regulatory circuits, to enable complex metabolic engineering. Streamlined Selection: Moving beyond antibiotic resistance to visual marker-free and high-throughput phenotyping systems for rapid identification of engineered lines, essential for multi-gene stacking and functional genomics.

### Unlocking fundamental biology for applied gains

Key biological unknowns currently limit full chassis optimization. Targeted research should focus on the cellular basis of hyper-growth by deciphering the cell cycle dynamics, meristem regulation, and secretory trafficking efficiency that underpin duckweed's unparalleled doubling rates; on developmental regulation through understanding the molecular mechanisms suppressing root and vascular development (body plan reduction) to reveal mechanisms that further simplify the chassis or control morphology; on dormancy diversity by expanding beyond the *Spirodela* turion model to enable better control over growth cycles and stress resilience in cultivation.

### Addressing practical challenges in industrial-scale cultivation

First, in high-density cultures, oxygen availability becomes a critical factor. Sun et al. (2023) demonstrated that under mixotrophic and heterotrophic conditions, duckweed cells enter a relatively low oxygen state, as evidenced by significant upregulation of fermentation pathway enzymes including pyruvate decarboxylase and alcohol dehydrogenase. To maintain optimal productivity, cultivation systems should incorporate gentle aeration or thin-film designs that maximize air–water interface without causing mechanical damage to fronds. Second, Duckweed harvesting presents unique challenges due to frond fragility and small size. Current methods fall into three categories, as reviewed by Ujong et al. (2025): mechanical skimming (conveyor belts or rotating drums, 60–80% efficiency), filtration systems (higher recovery > 90% but prone to clogging), and flocculation methods (high recovery but introducing chemical contaminants). Harvesting frequency significantly impacts productivity: Ujong et al. (2025) reported that harvesting twice per week yielded higher biomass (533 g/m^2^ fresh weight) compared to once-weekly harvesting (402 g/m^2^), with optimal intervals of 20 days producing the highest protein content (39.5%). These findings underscore that harvesting strategy must be optimized for each cultivation system and end-use application.

### Pioneering novel applications and scale-up

Finally, realizing duckweed's economic potential requires parallel advances in application and production by exploring new niches so that its native metabolism and edibility are leveraged to engineer high-value metabolites (flavonoids, terpenoids), oral enzymes, and nutraceuticals, creating "biomass-in-the-product" formats for functional feeds and foods; and by integrated process engineering such that monodisperse, sterile growth of duckweed can be maintained at industrial scale, integrating with upstream genetic engineering and downstream processing for end-to-end pilot-scale production. The feasibility of large-scale duckweed cultivation has been demonstrated by several initiatives. Sun et al. (2020) achieved remarkable productivity in mixotrophic conditions using 2 L bioreactors, with growth rates reaching 152.3 g/m^2^/day fresh weight, that is 4.98 and 6.22 times higher than in heterotrophic and photoautotrophic conditions, respectively. This translated to projected biomass yields of 49.6 t dry weight/ha/year. For starch production, Sun et al. (2023) reported that heterotrophic cultivation achieved starch content up to 44.9% of dry weight, while mixotrophic conditions balanced high biomass with 21.6% starch content. In wastewater treatment applications, Ujong et al. (2025) summarized studies showing duckweed effectively removes > 80% chemical oxygen demand (COD), > 90% total phosphorus, and > 50% total nitrogen from various waste streams, with biomass production simultaneously serving as feedstock for bioenergy or animal feed. These case studies collectively demonstrate that duckweed cultivation is technically feasible across diverse scales and applications, with productivity metrics that support commercial viability.

In conclusion, duckweed stands at a transformative threshold. By integrating deep biological insight with cutting-edge engineering principles, the scientific community can harness this minimalist plant to address maximalist challenges, from sustainable biomanufacturing and next-generation vaccines to carbon–neutral feed and beyond. The path forward is clear: unite systems-level data with robust genetic tools to program this versatile chassis, unlocking its full potential as a cornerstone of the global bioeconomy.

## Data Availability

Not applicable.
